# *Trichoderma viride* improves phosphorus uptake and the growth of *Chloris virgata* under phosphorus-deficient conditions

**DOI:** 10.3389/fmicb.2024.1425034

**Published:** 2024-07-04

**Authors:** Mingxia Song, Xiaoru Lin, Xiaowei Wei, Qingpan Zeng, Chunsheng Mu, Xiaofu Zhou

**Affiliations:** ^1^Key Laboratory of Vegetation Ecology of the Ministry of Education, Institute of Grassland Science, Northeast Normal University, Changchun, China; ^2^School of Life Sciences, Tonghua Normal University, Tonghua, China; ^3^Key Laboratory for Plant Resources Science and Green Production, Jilin Normal University, Siping, China

**Keywords:** *Trichoderma viride*, phosphorus uptake, phosphorus deficiency, *Chloris virgata*, growth

## Abstract

**Introduction:**

Phosphorus (P) readily forms insoluble complexes in soil, thereby inhibiting the absorption and utilization of this essential nutrient by plants. Phosphorus deficiency can significantly impede the growth of forage grass. While *Trichoderma viride* (*T. viride*) has been recognized for promoting the assimilation of otherwise unobtainable nutrients, its impact on P uptake remains understudied. Consequently, it is imperative to gain a more comprehensive insight into the role of *T. viride* in facilitating the uptake and utilization of insoluble P in forage grass.

**Methods:**

This research explored the influence of *T. viride* inoculation on P absorption and the growth of Chloris virgata (*C. virgata*) across various P sources. We treated plants with control P (P), tricalcium phosphate (TCP), calcium phytate (PHY), and low P (LP), with and without *T. viride* inoculation (P+T, TCP+T, PHY+T, LP+T). We analyzed photosynthesis parameters, growth indices, pigment accumulation, P content, leaf acid phosphatase activity.

**Results:**

Results demonstrated that *T. viride* inoculation alleviated inhibition of photosynthesis, reduced leaf acid phosphatase activity, and enhanced growth of *C. virgata* in the presence of insoluble P sources. Additionally, *T. viride* inoculation enabled the plants to extract more available P from insoluble P sources, as evidenced by a substantial increase in P content: shoot P content surged by 58.23 to 59.08%, and root P content rose by 55.13 to 55.2%. Biomass P-use efficiency (PUE) declined by 38% upon inoculation with *T. viride* compared to the non-inoculated insoluble P sources, paralleled by a reduction in photosynthetic P-use efficiency (PPUE) by 26 to 29%. Inoculation under insoluble P sources further triggered a lower allocation to root biomass (25 to 26%) and a higher investment in shoot biomass (74 to 75%). However, its application under low P condition curtailed the growth of *C. virgata*.

**Discussion:**

Our results suggest that *T. viride* inoculation represents an innovative approach for plants to acquire available P from insoluble P sources, thereby promoting growth amid environmental P limitations. This insight is crucial for comprehending the synergy among forage grass, P, and *T. viride*.

## Introduction

Phosphorus (P) is an essential macronutrient for plant growth and development, which plays a critical role in metabolism and protein regulation such as the synthesis of nucleotides, the composition of membranes, photosynthesis, respiration, and carbohydrate transportation (Zhang et al., 2014; Paz-Ares et al., 2022). Soil P occurs in various forms (organic and inorganic), which vary greatly in their bioavailability (Zhu et al., 2018). Plants predominantly utilize orthophosphate (Pi), which readily forms insoluble mineral complexes with ions such as Fe^3+^, Al^3+^, Ca^2+^, and Mg^2+^ within the soil, thus limiting P efficiency in fertilizers (Soltangheisi et al., 2018). Moreover, the phosphate rock used as the source material for P fertilizers is a finite and dwindling resource, which makes the possibility of a crisis realistic due to P scarcity at the end of this century (Sattari et al., 2012; Baker et al., 2015). Consequently, research on P efficiency mechanisms and plant sensitivity to P deficiency has emerged as a key scientific focus in recent years.

To adapt to P deficiency, plants have evolved various morphological and physiological traits to enhance P acquisition and P-use efficiency (PUE) (Lynch, 2011; Yang et al., 2024). Among these changes, the root system is the first part to perceive P stress, which plays an integral role in P uptake from the soil (Liu, 2021; Chen et al., 2023). To optimize P absorption, plants can adjust their root architecture, notably by reducing primary root elongation while promoting the growth of lateral roots and root hairs (Rellán-Álvarez et al., 2016; Motte et al., 2019). Root exudates, such as organic acids and phosphatases, have been shown to solubilize soil-bound insoluble P (Edayilam et al., 2018; Wang and Lambers, 2019). In addition, phosphate transporters (PHTs) play a critical role in regulating P uptake and distribution within plants (Victor Roch et al., 2019). Alterations in root–shoot signals coordinate P-deficient responses at the whole-plant level (Li et al., 2018). These changes prompt a cascade of physiological and biochemical reactions to enhance P absorption and redistribution among the different organs, tissues, cells, and organelles, affecting processes such as photosynthesis and carbohydrate transport (Wissuwa et al., 2005). Insufficient P supply to roots can lead to reduced leaf P content and potentially result in photoinhibition (Raven, 2011). Phosphorus deficiency has also been observed to decrease the net CO_2_ assimilation (A_n_) and stomatal conductance (g_s_) and inhibit photosystem II (PSII) activity and chlorophyll content, thereby inducing photoprotective mechanisms such as non-photochemical quenching (NPQ) and alternative electron transport pathways (Hernández and Munné-Bosch, 2015; Shi et al., 2020). Moreover, P deficiency triggers the top-down carbohydrate transport to support root P capture, leading to reduced shoot biomass and an increased root (R) to shoot (S) growth ratio (Lynch et al., 2005; Hu et al., 2021).

In adapting to P deficiency, the symbiotic relationship between plants and P-solubilizing microorganisms (PSMs) presents a more advantageous mechanism for plants to acquire P than direct uptake by roots alone (Qi et al., 2022). This symbiosis requires fewer plant resources compared to the mechanism of direct absorption (Etesami et al., 2021). Moreover, PSMs dissolve insoluble P by secreting organic acids and enzymes, facilitating P uptake by plants. In return, PSMs derive organic nutrients and photosynthates from their host plants (Smith and Smith, 2011). Notably, fungi of the genus *Trichoderma* have shown an ability to solubilize insoluble P and establish beneficial symbiotic relationships with plants (Duan et al., 2023). Wang et al. (2018) observed that *T. viride* biofertilizer reduced NH_3_ volatilization in alkaline soil and enhanced the growth of sweet sorghum. Garstecka et al. (2023) discovered that *T. viride* could colonize roots and increase the yield of *Canola* under drought conditions. Metwally and Soliman (2023) found that *T. viride* inoculation alleviated the salt stress of tomato seedlings and maintained their normal growth. Our previous experiments have confirmed that *T. viride* can solubilize insoluble P and alter the root morphology of legume grasses (Song et al., 2023). Extending previous work in this field, our current study delves into the regulatory effects of *T. viride* on gramineous grasses under P deficiency, examining physiological and biochemical responses and P distribution in depth.

The *Chloris virgata* is a tufted annual grass of the Gramineae family, renowned for its high nutritional value and excellent stress resistance as a forage. P is very important for the growth of forage grasses. However, studies investigating the impact of P deficiency on *C. virgata* are limited. In this study, *C. virgata* was exposed to different P sources to evaluate the effect of *T. viride* inoculation on its P uptake efficiency and growth. Three critical questions were discussed throughout this study: (1) Does *T. viride* inoculation positively influence the photosynthetic characteristics of *C. virgata* under P deficiency? (2) Does *T. viride* inoculation enhance the P uptake and P-use efficiency in *C. virgata*? (3) What impact does *T. viride* inoculation have on the growth and biomass allocation of *C. virgata*?

## Materials and methods

### Materials and growth conditions

The *Trichoderma viride* strain, isolated from Ginseng in Jilin Province, China, has been securely deposited at the China General Microbiological Culture Collection Center (CGMCC; No.40034) to ensure its sustained availability for research purposes. Spores were induced to germinate on potato dextrose agar medium, maintained at a stable temperature of 28°C for a period of 7 days. Following germination, spore suspensions were crafted using sterile water augmented with 0.01% Tween-80, and the concentration was adjusted to 1 × 10^7^ cfu L^−1^ using a hemocytometer.

The seedlings of *C. virgata* which had been cultivated for 15 days were selected for this study. Seedlings were transplanted into the plastic pots (17 cm diameter × 15 cm depth), each filled with sterilized vermiculite (0.3 kg pot^−1^) and irrigated with a 1/4 strength of Hoagland nutrient solution (Hoagland and Arnon, 1950). The nutrient solution was prepared at two levels of P concentration: 1000 μM KH_2_PO_4_ (control P) and 10 μM KH_2_PO_4_ (low P). For the latter, KCl was used to replace KH_2_PO_4_, maintaining a consistent potassium (K^+^) concentration of 6 mM across all treatments. Tricalcium phosphate (TCP) and calcium phytate (PHY) were selected as insoluble P sources in the experiment.

The experiment consisted of eight treatments: (1) P: 1000 μM KH_2_PO_4_, (2) P + T: 1000 μM KH_2_PO_4_ with *T. viride* spore suspensions, (3) TCP: 10 μM KH_2_PO_4_ and tricalcium phosphate, (4) TCP + T: 10 μM KH_2_PO_4_ and tricalcium phosphate with *T. viride* spore suspensions, (5) PHY: 10 μM KH_2_PO_4_ and calcium phytate, (6) PHY + T: 10 μM KH_2_PO_4_ and calcium phytate with *T. viride* spore suspensions, (7) LP: 10 μM KH_2_PO_4_, and (8) LP + T: 10 μM KH_2_PO_4_ with *T. viride* spore suspensions. The content of total P used in each treatment was 27.64 mg. There were six pots per treatment, and one pot served as a biological repeat. For each pot, 180 mL of nutrient solution was added to vermiculite every 6 days. In the treatments with *T. viride* inoculation, the spore suspensions were applied to the seedlings with 60 mL every 15 days. The experiment was conducted in a greenhouse, where seedlings were cultivated for 30 days at 27°C day/23°C night temperature, under a 16-h light/8-h dark photoperiod, with 680 μmol m^−2^ s^−1^ irradiance and 40% relative humidity. After the growing period, seedlings were harvested for physiological and biochemical assays.

### Gas exchange measurements and chlorophyll fluorescence

The gas exchange parameters were measured on the second leaf using a CIRAS-3 portable photosynthesis system (PP Systems, United States) at 25°C, relative humidity of 65%, a cuvette air flow rate of 500 μmol s^−1^, and a CO_2_ concentration at 400 μmol mol^−1^. The photosynthetic photon flux density (PPFD) was set to 1,600 μmol m^−2^ s^−1^ with a combination of 90% red light, 5% blue light, and 5% white light and measured between 8:00 a.m. and 12:00 a.m. (six replicates). The parameters include CO_2_ assimilation rate (A_n_, μmol m^−2^ s^−1^), stomatal conductance (g_s_, mmol m^−2^ s^−1^), and internal CO_2_ (C_i_, mmol mol^−1^).

IMAGING-PAM M-series (Walz, Effeltrich, Germany) was used to measure chlorophyll fluorescence, and all samples were kept in a dark period of 20 min before measurements. The fluorescence parameters include the effective quantum yield of PSII (ΦPSII), the maximum quantum yield of PSII (Fv/Fm), non-photochemical quenching coefficient (NPQ), and electron transport rate (ETR, μmol e^−1^ s^−1^ m^−2^), which were calculated according to the relevant study (Zhou et al., 2021).

### The determination of growth indices

The growth was evaluated by measuring the morphological characteristics of the roots and plant dry weight (DW). The fresh roots were scanned by a desktop scanner (EPSON Perfection V 700 Photo; Epson, America, Inc., United States) and analyzed by the WinRHIZO image analysis system (WinRHIZO 2012 b; Regent, Canada). Then, the parameters of the root morphology characteristics were obtained, such as total root length (cm), root surface area (cm^2^), and root volume (cm^3^). The fresh shoots (S) and roots (R) were dried at 105°C for 30 min and then continued drying at 70°C for 10 h, respectively. The root-to-shoot growth ratio was calculated as follows: R/S ratio = root dry weight/shoot dry weight.

### Determination of leaf photosynthetic pigment

Fresh leaves of 0.2 g were taken as a sample and immersed in 10 mL of 95% (v/v) ethanol for 24 h. The extracts were then diluted 3-fold with 95% (v/v) ethanol, and their absorbance was measured using a spectrophotometer (Hitachi U-3000; Hitachi, Ltd., Chiyoda, Tokyo, Japan) at 470 nm, 649 nm, and 665 nm. The extract absorbances of Chl a, Chl b, Chl a + b, and Car were calculated according to Wellburn and Lichtenthaler (1984). Pigment content was calculated as follows (C represents Chl a, Chl b, Chl a + b, and Car, V is the extract volume, T is the dilution multiple, and M is the leaf weight):

$$Chl\;a=13.95\times A_{665}-6.88\times A_{649}\text{,}$$

$$Chl\;b=24.96\times A_{649}-7.32\times A_{665}\text{,}$$

Chla+b=Chla+Chlb,

$$\mathrm{Car}=\left(1000\times A_{470}-2.05\times Chl\;a-114.8\times Chl\;b\right)/245,\mathrm{and}$$

$$\mathrm{Pigment}\;\mathrm{content}\;\left(\mathrm{mg}\;g^{-1}\right)=\frac{C\times V\times T}{M\times 1000}$$

### Determination of P content

The total P content in both shoots and roots was measured by the molybdenum blue method (Murphy and Riley, 1962). The samples were dried at 105°C for 30 min followed by a prolonged drying at 65°C for 10 h. Then, 1 g of the dried samples was digested in a mixed-acid solution with a volumetric ratio of 8:1:1 (HNO_3_:HClO_4_:H_2_SO_4_) to facilitate the quantification.

### Determination of leaf acid phosphatase activity

The activity of acid phosphatase (ACP) was evaluated using a plant enzyme activity assay kit (Shanghai MLBIO Biotechnology Co., Ltd., China). The leaf samples were quantified by 0.1 g and then ground on the ice in 1 mL extraction buffer. The homogenate was centrifuged at 10000 × *g* at 4°C for 10 min. The supernatant was used in the enzyme activity assays. ACP activity was evaluated by measuring the change in absorbance at 405 nm.

### The calculation of P-use efficiency and biomass resource allocation

Two metrics of P-use efficiency were calculated: biomass P-use efficiency (PUE), which pertains to biomass production, and photosynthetic P-use efficiency (PPUE), which relates to the rate of C fixation per unit leaf P. Each parameter offers insights into distinct aspects of P utilization: PUE provides a measure at the whole-plant level, while PPUE gauges efficiency at the photosynthetic level. The PUE and PPUE were calculated as follows (Hayes et al., 2022):

$$PUE=\frac{\mathrm{Total}\;\mathrm{Biomass}}{\mathrm{Total}\;P\;}$$

$$\mathrm{PPUE}=\frac{\mathrm{An}}{\mathrm{Leaf}\;P\times LMA}\;\text{.}$$

The leaf mass per unit area (LMA) in the formula is calculated in Supplementary Table S1.

The resource allocation of biomass was calculated as follows:

$$\mathrm{Shoot}\;DW\left(\%\right)=\frac{\mathrm{Shoot}\;DW}{\mathrm{Total}\;DW}\times 100\%$$

$$\mathrm{Root}\;DW\left(\%\right)=\frac{\mathrm{Root}\;DW}{\mathrm{Total}\;DW}\times 100\%\text{.}$$

### Statistical analysis

The experiment utilized a completely randomized design with six replicates per treatment. To determine the impact of different P treatments and *T. viride* inoculation on plant parameters, one-way ANOVA was conducted, followed by Duncan’s test (*p* < 0.05). The effects between P treatments, *T. viride* inoculation, and their interaction (P × T) were examined using two-way ANOVA and also assessed with Duncan’s test (*p* < 0.05). All statistical analyses were performed using the SPSS statistical software 26.0. Figures were created with OriginPro 2021.

## Results

### Leaf photosynthetic characteristic response to P deficiency

**Figure 1:**
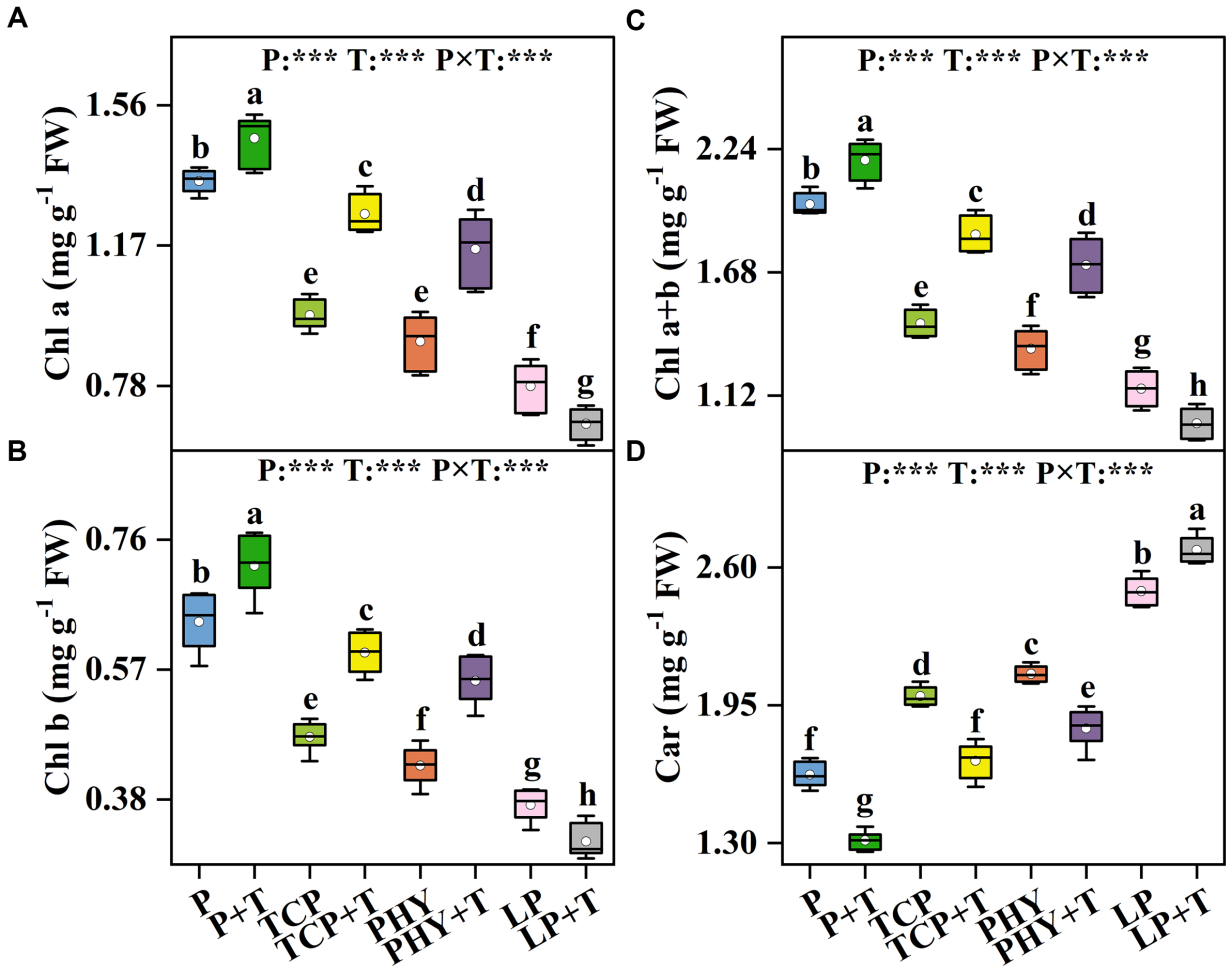
Effect of P-deficient treatments on chlorophyll a (Chl a) **(A)**, chlorophyll b (Chl b) **(B)**, total chlorophyll (Chl a + b) **(C)**, and carotenoid (Car) **(D)**. P, P was added as KH_2_PO_4_; TCP, P was added as tricalcium phosphate; PHY, P was added as calcium phytate; LP, P was added as 10 μM L^−1^ KH_2_PO_4_. +T, with *Trichoderma viride* inoculation. The white dot is “Mean”; the black diamond shape is “Outlier”; the horizontal is “Median”; the top of the vertical line is “Max”; and the bottom of the vertical line is “Min.” Different lowercase letters indicate significant differences in Duncan’s test (*p* < 0.05) (*n* = 6). ^***^indicates a significant difference extremely (*p* < 0.001).

The Chl a, Chl b, Chl a + b, and Car contents in *C. virgata* treated with P, T, and their interaction (P × T) displayed statistically significant variations (*p* < 0.001) (Figure 1). Compared to the non-inoculated treatments, *T. viride* inoculation led to an increase in Chl a, Chl b, and Chl a + b contents, except for the LP + T treatment (Figures 1A–C). In the LP + T treatment, Chl a, Chl b, and Chl a + b contents were reduced by 13.44, 14.30, and 13.71%, respectively, in comparison to the LP treatment. Conversely, the TCP + T treatment enhanced Chl a, Chl b, and Chl a + b contents by 28.84, 26.13, and 27.96%, respectively, over the TCP treatment. Similar increases were observed in the PHY + T treatment, with Chl a, Chl b, and Chl a + b contents increased by 28.41, 28.93, and 28.56%, respectively, in comparison with the PHY treatment. The Car contents of the treatments with *T. viride* inoculation were lower compared to non-inoculated *T. viride*, except for the LP + T treatment, which presented a 7.8% increase over the LP treatment (Figure 1D). The TCP + T and PHY + T treatments displayed a reduction in Car content by 16.3 and 12.2%, respectively, compared to their corresponding non-inoculated treatments.

**Figure 2:**
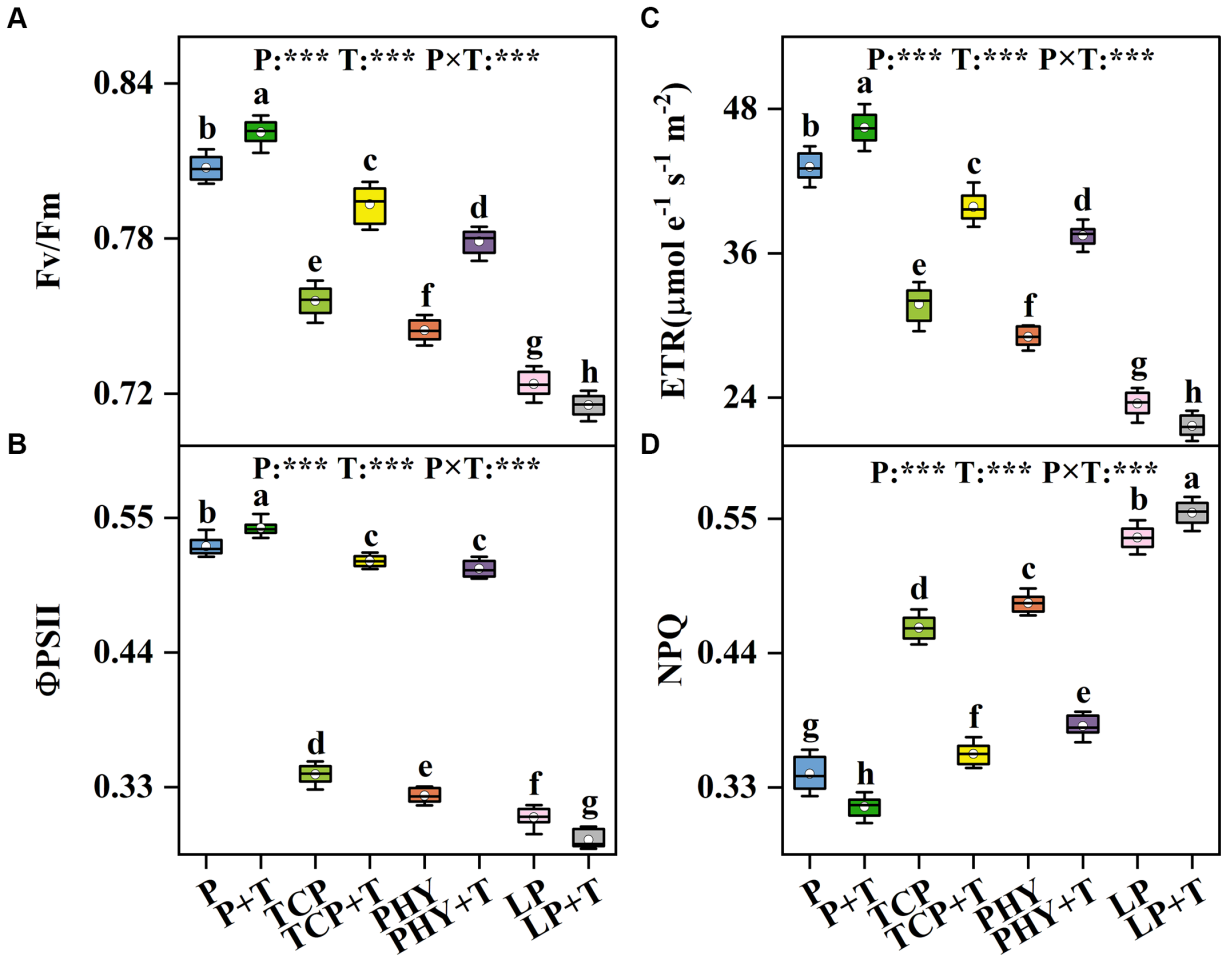
Effect of P-deficient treatments on the maximum quantum yield of PSII (Fv/Fm) **(A)**, the effective quantum yield of PSII (ΦPSII) **(B)**, electron transport rate (ETR) **(C)**, and non-photochemical quenching coefficient (NPQ) **(D)**. P, P was added as KH_2_PO_4_; TCP, P was added as tricalcium phosphate; PHY, P was added as calcium phytate; LP, P was added as 10 μM L^−1^ KH_2_PO_4_. +T, with *Trichoderma viride* inoculation. The white dot is “Mean”; the black diamond shape is “Outlier”; the horizontal is “Median”; the top of the vertical line is “Max”; and the bottom of the vertical line is “Min.” Different lowercase letters indicate significant differences in Duncan’s test (*p* < 0.05) (*n* = 6). ^***^indicates a significant difference extremely (*p* < 0.001).

The chlorophyll fluorescence parameters in *C. virgata* treated with P, T, and their interaction (P × T) displayed statistically significant variations (*p* < 0.001) (Figure 2). Compared to the non-inoculated treatments, the *T. viride* inoculation increased the values of Fv/Fm, ΦPSII, and ETR, except for the LP + T treatment (Figures 2A–C). In the LP + T treatment, Fv/Fm, ΦPSII, and ETR values were reduced by 1.14, 6.02, and 7.94%, respectively, compared to the LP treatment. However, the TCP + T treatment enhanced Fv/Fm, ΦPSII, and ETR values by 4.93, 51.2, and 25.51%, respectively, over the TCP treatment. Similar increases were observed in the PHY + T treatment, with Fv/Fm, ΦPSII, and ETR values increased by 4.62, 57.3, and 29.03%, respectively, in comparison with the PHY treatment. Conversely, the NPQ values of the treatments with *T. viride* were lower compared to non-inoculated *T. viride*, except for the LP + T treatment, which presented a 3.80% increase over the LP treatment (Figure 2D). The TCP + T and PHY + T treatments displayed a reduction in NPQ value by 22.42 and 20.93%, respectively, compared to their corresponding non-inoculated treatments.

**Figure 3:**
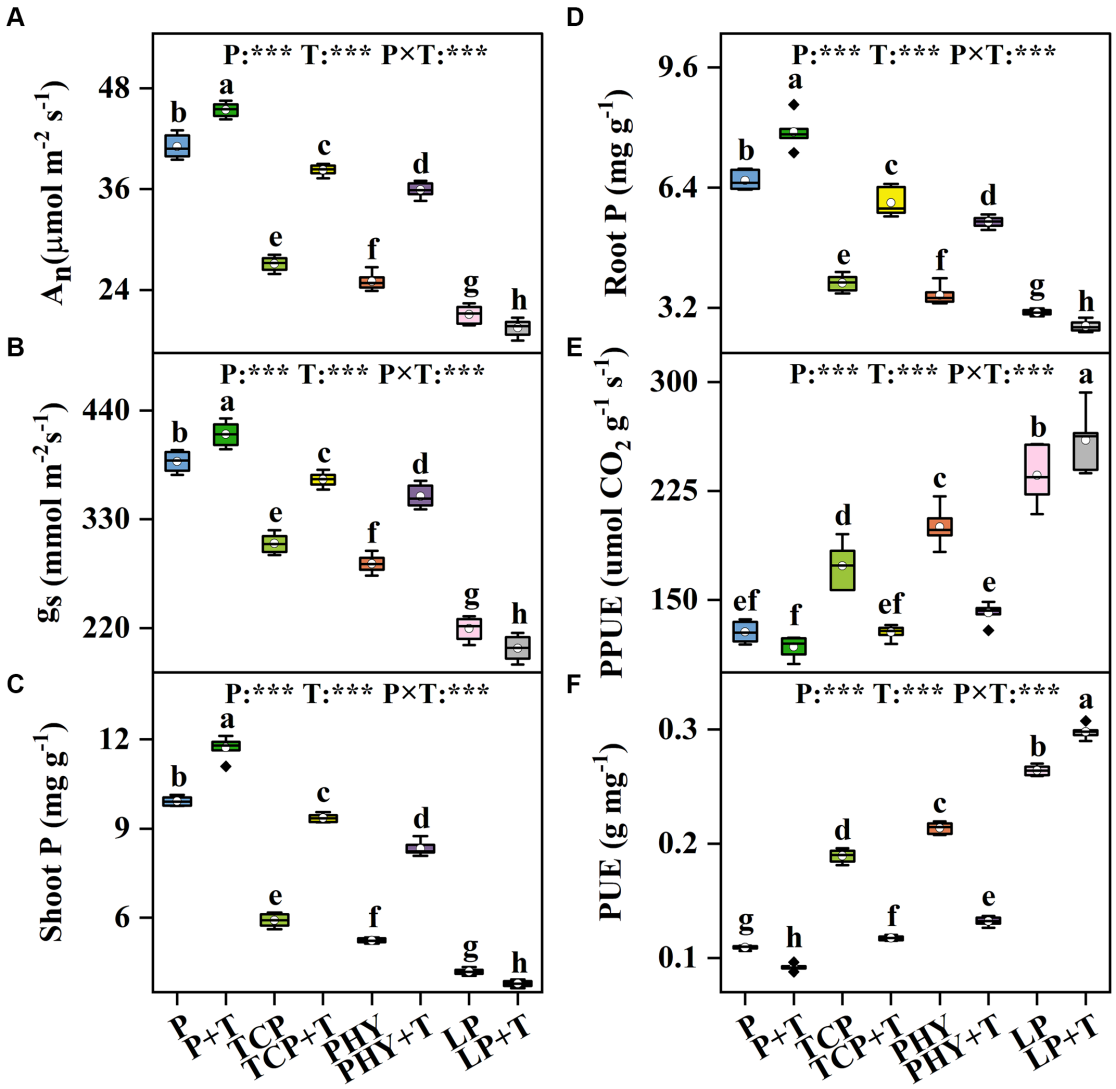
Effect of P-deficient treatments on CO_2_ assimilation rate (A_n_) **(A)**, stomatal conductance (g_s_) **(B)**, shoot P content (shoot P) **(C)**, root P content (root P) **(D)**, photosynthetic P-use efficiency (PPUE) **(E)**, and biomass P-use efficiency (PUE) **(F)**. P, P was added as KH_2_PO_4_; TCP, P was added as tricalcium phosphate; PHY, P was added as calcium phytate; LP, P was added as 10 μM L^−1^ KH_2_PO_4_. +T, with *Trichoderma viride* inoculation. The white dot is “Mean”; the black diamond shape is “Outlier”; the horizontal is “Median”; the top of the vertical line is “Max”; and the bottom of the vertical line is “Min.” Different lowercase letters indicate significant differences in Duncan’s test (*p* < 0.05) (*n* = 6). ^***^indicates a significant difference extremely (*p* < 0.001).

The A_n_ and g_s_ in *C. virgata* treated with P, T, and their interaction (P × T) displayed statistically significant variations (*p* < 0.001) (Figures 3A,B). Compared to the non-inoculated treatments, the *T. viride* inoculation significantly increased the values of A_n_ and g_s_, except for the LP + T treatment (Figures 3A,B). Specifically, A_n_ and g_s_ values in the LP + T treatment were 7.58 and 9.04% lower compared to the LP treatment, respectively. However, the TCP + T treatment enhanced A_n_ and g_s_ values by 41.18 and 21.17%, respectively, over the TCP treatment. Similar increases were observed in the PHY + T treatment, with A_n_ and g_s_ values increased by 43.57 and 23.92%, respectively, in comparison with the PHY treatment. Conversely, the C_i_ under P treatment showed highly significant differences (*p* < 0.001). However, the T and P × T treatments did not significantly affect C_i_ (Supplementary Figure S1). The trend in C_i_ values was inversely related to A_n_ and g_s_, with the LP + T treatment exhibiting a 6.06% increase in C_i_ over the LP treatment (Supplementary Figure S1). The TCP + T and PHY + T treatments displayed a reduction in C_i_ value by 27.88 and 23.57%, respectively, compared to their corresponding non-inoculated treatments.

### P uptake and P-use efficiency

The shoot and root P contents in *C. virgata* treated with P, T, and their interaction (P × T) displayed statistically significant variations (*p* < 0.001) (Figures 3C,D). Compared to the non-inoculated treatments, the *T. viride* inoculation significantly increased shoot P contents, except for the LP + T treatment, which presented a 9.6% decline over the LP treatment (Figure 3C). A similar trend was observed for root P contents, with the LP + T treatment exhibiting an 11.95% decrease in root P content over the LP treatment (Figure 3D). Then, the TCP + T treatment enhanced shoot P and root P contents by 58.23 and 55.13%, respectively, over the TCP treatment. Similar increases were observed in the PHY + T treatment, with shoot P and root P contents increased by 59.08 and 55.2%, respectively, in comparison with the PHY treatment.

The PPUE and PUE in *C. virgata* treated with P, T, and their interaction (P × T) displayed statistically significant variations (*p* < 0.001) (Figures 3E,F). Compared to the non-inoculated treatments, the *T. viride* inoculation significantly decreased the PPUE and PUE values, except for the LP + T treatment (Figures 3E,F). The PPUE and PUE values in the LP + T treatment were 10 and 12.8% higher compared to the LP treatment, respectively. Conversely, the TCP + T treatment decreased PPUE and PUE values by 26 and 38%, respectively, over the TCP treatment. Similar decreases were observed in the PHY + T treatment, with PPUE and PUE values reduced by 29 and 38.22%, respectively, in comparison with the PHY treatment.

### Leaf P assimilation enzyme activity

**Figure 4:**
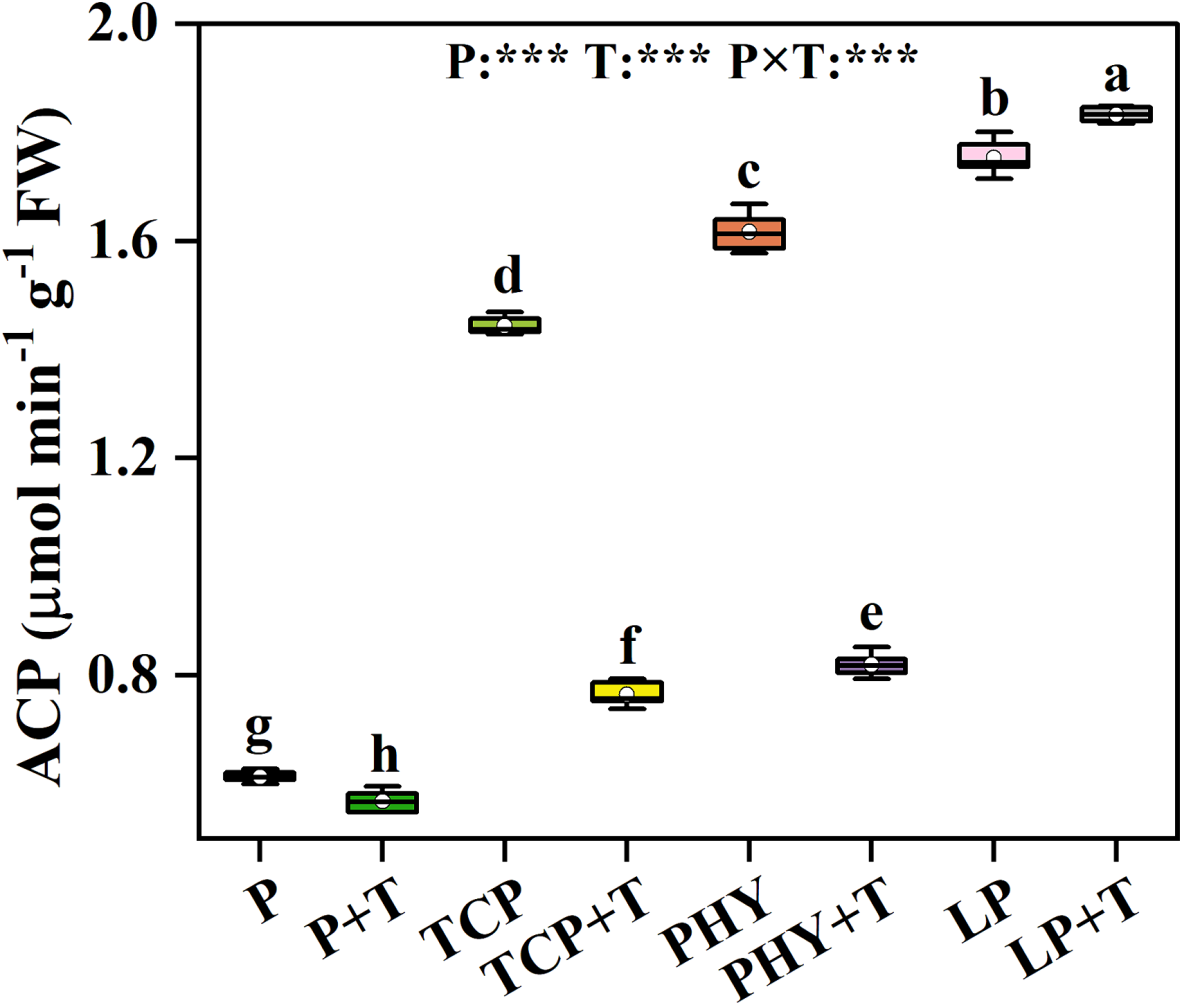
Effect of P-deficient treatments on acid phosphatase (ACP) activity. P, P was added as KH_2_PO_4_; TCP, P was added as tricalcium phosphate; PHY, P was added as calcium phytate; LP, P was added as 10 μM L^−1^ KH_2_PO_4_. +T, with *Trichoderma viride* inoculation. The white dot is “Mean”; the black diamond shape is “Outlier”; the horizontal is “Median”; the top of the vertical line is “Max”; and the bottom of the vertical line is “Min.” Different lowercase letters indicate significant differences in Duncan’s test (*p* < 0.05) (*n* = 6). ^***^indicates a significant difference extremely (*p* < 0.001).

The ACP activity in *C. virgata* treated with P, T, and their interaction (P × T) displayed statistically significant variations (*p* < 0.001) (Figure 4). Compared to the non-inoculated treatments, the *T. viride* inoculation significantly reduced ACP activity, except for the LP + T treatment (Figure 4). In the LP + T treatment, ACP activity increased by 4.54% compared to the LP treatment alone. The ACP activity in the TCP + T and the PHY + T treatments reduced by 47.04 and 49.31%, respectively, compared to the TCP and PHY treatments without *T. viride* inoculation.

### Growth and biomass resource allocation

**Figure 5:**
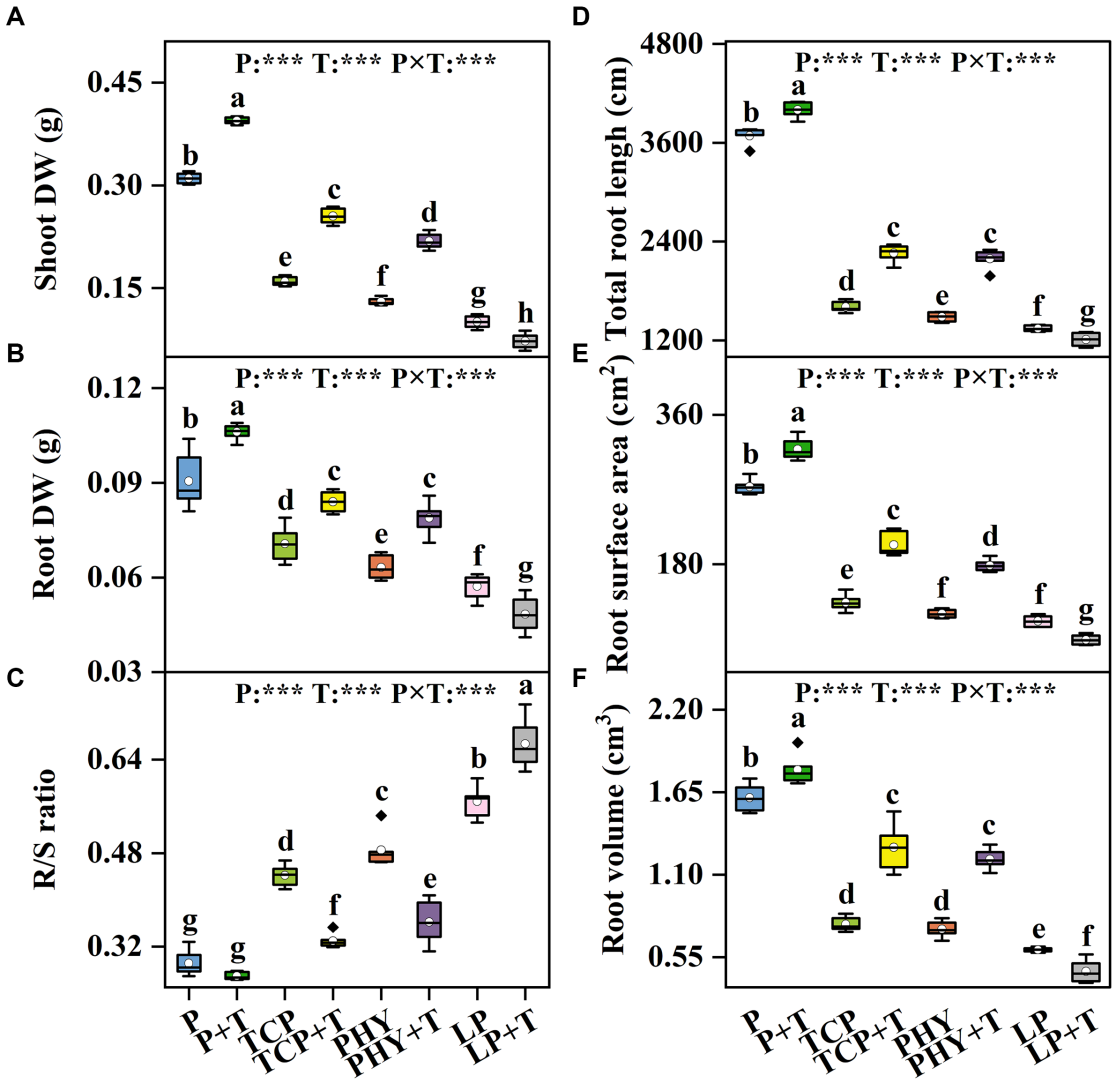
Effect of P-deficient treatments on shoot dry weight (shoot DW) **(A)**, root dry weight (root DW) **(B)**, root-to-shoot growth ratio (R/S ratio) **(C)**, total root length **(D)**, root surface area **(E)**, and root volume **(F)**. P, P was added as KH_2_PO_4_; TCP, P was added as tricalcium phosphate; PHY, P was added as calcium phytate; LP, P was added as 10 μM L^−1^ KH_2_PO_4_. +T, with *Trichoderma viride* inoculation. The white dot is “Mean”; the black diamond shape is “Outlier”; the horizontal is “Median”; the top of the vertical line is “Max”; and the bottom of the vertical line is “Min.” Different lowercase letters indicate significant differences in Duncan’s test (*p* < 0.05) (*n* = 6). ^***^indicates a significant difference extremely (*p* < 0.001).

The shoot DW, root DW, and R/S ratio in *C. virgata* treated with P, T, and their interaction (P × T) displayed statistically significant variations (*p* < 0.001) (Figures 5A–C). Compared to the non-inoculated treatments, the *T. viride* inoculation significantly increased shoot DW and root DW values, except for the LP + T treatment, which was 27.77 and 15.45% lower, respectively, compared to the LP treatment (Figures 5A,B). However, the TCP + T treatment enhanced shoot DW and root DW values by 59.65 and 18.9%, respectively, over the TCP treatment. Similar increases were observed in the PHY + T treatment, with shoot DW and root DW values increased by 67.77 and 24.8%, respectively, in comparison with the PHY treatment. The R/S ratio in the TCP + T treatment decreased by 25.4% compared to TCP, aligning with the values observed for the PHY and PHY + T treatments (Figure 5C). Conversely, the R/S ratio in the LP + T treatment increased by 17.4% compared to the LP treatment.

The parameters of the root morphology characteristics in *C. virgata* treated with P, T, and their interaction (P × T) displayed statistically significant variations (*p* < 0.001) (Figures 5D–F). Compared to the non-inoculated treatments, the *T. viride* inoculation significantly increased the values of total root length, root surface area, and root volume, except for the LP + T treatment, which showed reductions of 9.99, 20.13, and 24.17% in these parameters compared to the LP treatment alone (Figures 5D–F). However, the TCP + T treatment enhanced total root length, root surface area, and root volume values by 41, 52, and 66.45%, respectively, over the TCP treatment. Similar increases were observed in the PHY + T treatment, with total root length, root surface area, and root volume values increased by 48, 48, and 63.35%, respectively, in comparison with the PHY treatment. Among all the treatments, the P + T treatment yielded the highest values for these root parameters.

**Figure 6:**
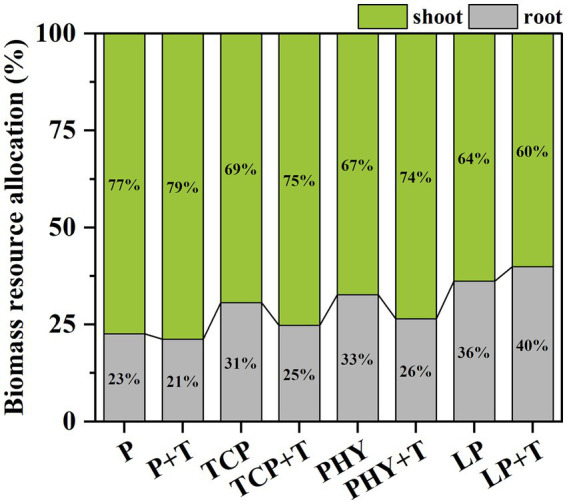
Effect of P-deficient treatments on biomass resource allocation. P, P was added as KH_2_PO_4_; TCP, P was added as tricalcium phosphate; PHY, P was added as calcium phytate; LP, P was added as 10 μM L^−1^ KH_2_PO_4_. +T, with *Trichoderma viride* inoculation. The green part is the proportion of shoot dry weight to total biomass; the gray part is the proportion of root dry weight to total biomass.

Ultimately, the *C. virgata* modified its resource allocation when inoculated with *T. viride.* Compared to the non-inoculated treatments, the *T. viride* inoculation resulted in a significant increase in shoot biomass allocation and a corresponding reduction in root biomass allocation, except for the LP + T treatment (Figure 6). The P + T treatment displayed the greatest resource allocation to shoot growth, whereas the LP + T treatment had the lowest among all treatments. In the TCP + T treatment, plants directed 75% more resources to their shoot biomass and allocated only 25% to root biomass. A similar pattern was noted in the PHY + T treatment, with plants allocating 74% more to shoot biomass and only 26% to root biomass.

### Correlation analysis with P content and A_n_ and PPUE and biomass and PUE

**Figure 7:**
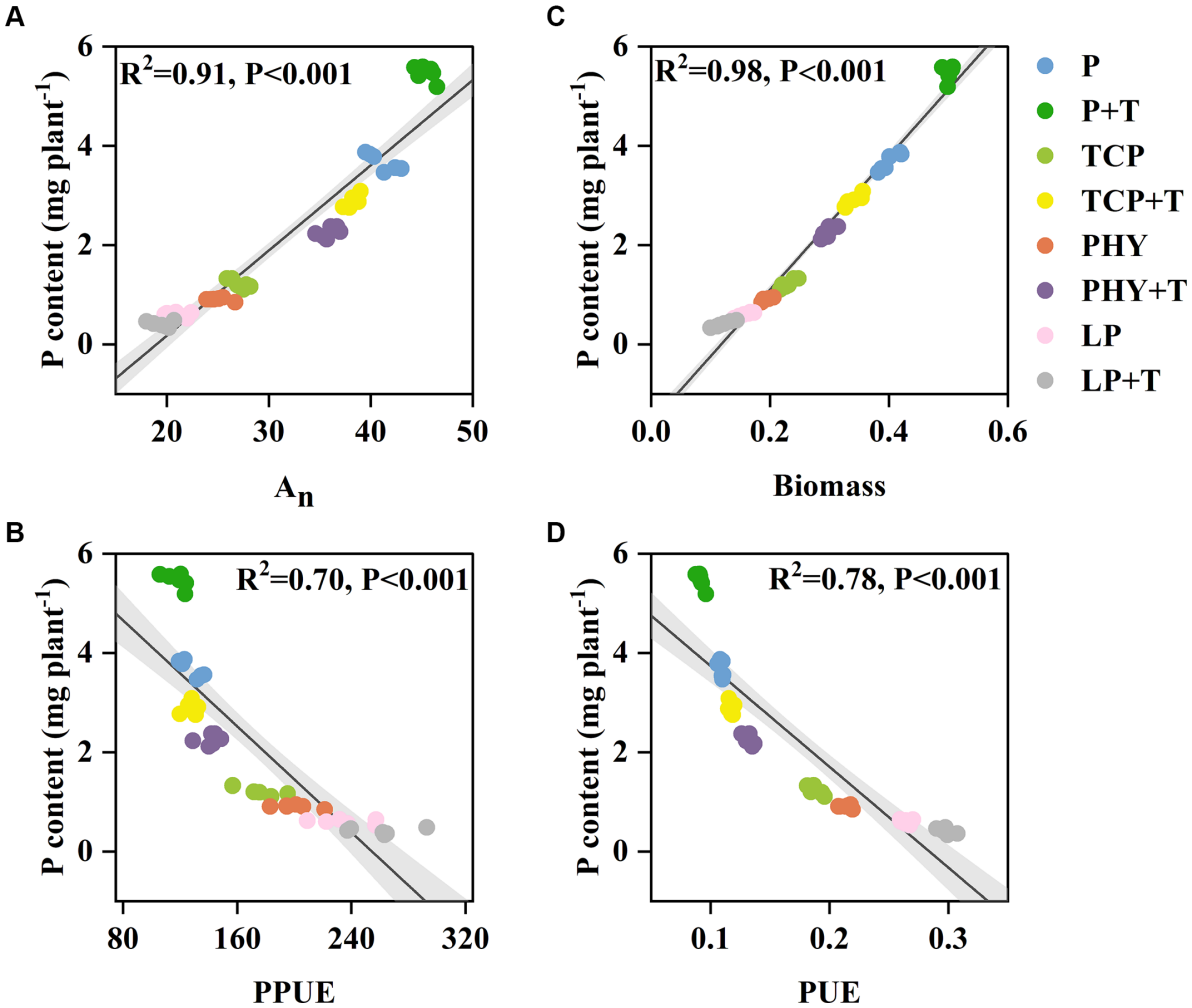
Relationships between P content and CO_2_ assimilation rate (A_n_) **(A)**, photosynthetic P-use efficiency (PPUE) **(B)**, biomass **(C)**, and biomass P-use efficiency (PUE) **(D)**. P, P was added as KH_2_PO_4_; TCP, P was added as tricalcium phosphate; PHY, P was added as calcium phytate; LP, P was added as 10 μM L^−1^ KH_2_PO_4_. +T, with *Trichoderma viride* inoculation. R^2^ values for linear trend lines are shown on each plot (*p* < 0.05, significant) (*n* = 6).

The results indicated a positive correlation between plant P content and A_n_ (Figure 7A). Conversely, plant P content had a negative correlation with PPUE (Figure 7B). There was a significant positive association between plant P content and biomass (Figure 7C), whereas plant P content had a significant negative relationship with PUE (Figure 7D).

## Discussion

Phosphorus is an indispensable nutrient critical for plant development, and its deficiency typically hinders growth. Currently, harnessing P-solubilizing microorganisms offers a novel approach to facilitate plant absorption of insoluble soil P. Consequently, discerning plant biological responses to diverse P supplies facilitated by these microorganisms has become imperative. In our study, we conducted a suite of experiments to examine the regulatory effects of *T. viride* inoculation on *C. virgata* (a C_4_ plant) cultivated in varying P conditions. Our findings unequivocally demonstrate that *T. viride* inoculation enhanced photosynthetic characteristics, P uptake, and P-use efficiency. Moreover, it altered biomass allocation and promoted the growth of *C. virgata* in environments with either insufficient inorganic soluble P or insoluble forms of P.

### Effects of the *T. viride* inoculation on photosynthesis under P deficiency

P-solubilizing microorganisms are recognized for their ability to transform insoluble P into an absorbable and utilizable form for plants (Pang et al., 2024). While symbiotic associations with PSMs typically confer advantages to plants, PSMs may impede growth under conditions of no P or extreme P scarcity (Qi et al., 2022). Phosphorus deficiency often results in photosystem alterations, leading to photo-oxidative stress (Lin et al., 2009). Specifically, it diminishes chlorophyll content, adversely affects light-harvesting capabilities, and subsequently depresses the Fv/Fm of PSII (Carstensen et al., 2018b; Xia et al., 2023). Our study revealed that *T. viride* inoculation significantly increased Chl a, Chl b, and Chl a + b contents in treatments with insoluble P, while the lowest contents were observed in the LP + T treatment. Moreover, P deficiency increases Car content (Li et al., 2018), a finding our research corroborates, potentially due to the involvement of the xanthophyll cycle. Under P-deficient conditions, leaf assimilation ability and ATP synthesis suffer notable declines, causing reduced electron transport rate, Fv/Fm, and ΦPSII (Carstensen et al., 2018a). Consistently, our result indicated significant decreases in ETR, Fv/Fm, and ΦPSII under LP and insoluble P treatments, whereas *T. viride* inoculation generally led to improvements, except in the LP + T treatment. Facing P deficiency, plants have evolved various adaptive mechanisms to mitigate photo-oxidative damage. NPQ, the first line of defense against photo-oxidative stress that dissipates excess energy as heat, increased under P-deficient conditions (Guidi et al., 2019; Xia et al., 2021). Correspondingly, our findings showed a considerable increase in NPQ in LP and insoluble P treatments, apart from *T. viride* inoculation, excluding the LP + T treatment. Therefore, we speculate that *C. virgata* with *T. viride* inoculation can maintain the normal operation of the photosynthetic apparatus under insoluble P conditions by enhancing pigment-driven light capture and PSII activity. However, *T. viride* inoculation in low P conditions resulted in diminished photosynthetic performance. This reduction could be attributed to a disrupted P redistribution mechanism, stemming from competition for limited P between *T. viride* and *C. virgata*, potentially causing an imbalance between the plant’s capacity for processing light energy and the incoming light levels.

Studies indicated that the decrease in the A_n_ under P deficiency was not solely due to a decline in g_s_ as C_i_ usually showed an increase in most cases (Hernández and Munné-Bosch, 2015; Li et al., 2018). Further detailed studies have identified non-stomatal limitations as the primary constraints on A_n_ in conditions of P deficiency (Singh and Reddy, 2011; Warren, 2011). In our study, A_n_ and g_s_ decreased, while C_i_ increased in response to P deficiency. On the contrary, *T. viride* inoculation typically produced the reverse effects, except for the LP + T treatment. In addition, A_n_ was positively correlated with enhanced P concentration. Hence, we infer that *T. viride* inoculation in the presence of insoluble P can generate more carbohydrates and mitigate adverse P stress impacts on *C. virgata*.

### Effects of the *T. viride* inoculation on P uptake and P-use efficiency under P deficiency

Our study demonstrated that both the shoot and the root P contents declined significantly under the insoluble P treatments, while *T. viride* inoculation led to an increase in these parameters. Notably, under insoluble P conditions, the shoot P content exceeded that of the roots. Corresponding with the findings from previous research, P deficiency precipitated a decrease in the P content of both shoots and roots, yet the shoot tended to accumulate more P resources than the roots (Plenet et al., 2000; Görlach and Mühling, 2021; Yang et al., 2024). This phenomenon could be attributed to the mitigating effect of *T. viride* inoculation on P deficiency in *C. virgata*, especially under insoluble P conditions, which in turn facilitates a greater allocation of P to the aboveground parts, thereby enhancing photosynthate production.

Plants have high PPUE in severely P-impoverished soil because plants allocate more P to mesophyll cells to maintain photosynthesis under P deficiency (Hidaka and Kitayama, 2013). However, P-rich mesophyll cells only represent a small part of the whole leaf, which are effectively diluted by the large portion of P-poor non-photosynthetic cells in the leaf (Hayes et al., 2018). Thus, plants maintain high PPUE even with markedly low leaf phosphorus levels. Consistent with this, our analysis revealed a significant increase in PPUE as P availability declined and a negative correlation with leaf P content, indicating an adaptive strategy in response to P deficiency. The PUE reflects the whole-plant level P-use efficiency (Hayes et al., 2022). Our study showed that PUE increased with decreasing P availability and was correlated negatively with increased P content. The *T. viride* inoculation reduced PUE in all treatments, except for the LP + T treatment. Consequently, this implies that *C. virgata* can sustain considerable biomass production despite P deficiency. However, *T. viride* inoculation boosted both biomass and plant P content, with a more pronounced increase in plant P content under conditions of P sufficiency.

Acid phosphatase is a crucial enzyme for hydrolyzing organic P into orthophosphate, and it is secreted by plants in response to P deficiency (Yan et al., 2001). Within the cell, this enzyme converts organic P in aged leaves into orthophosphate, which is then transported to younger leaves when P is scarce (Snapp and Lynch, 1996). The acid phosphatase activity showed an increase in leaves under P deficiency, compared with P sufficiency (Zhang et al., 2010). Our study observed a similar pattern of intracellular acid phosphatase activity. Furthermore, the *T. viride* inoculation can mitigate the overactivation of acid phosphatase by satisfying the plant’s P requirements. Consequently, *T. viride* inoculation under conditions of insoluble P can curtail the plant’s investment in P assimilation enzymes, thereby reallocating resources to bolster growth.

### The effects of the *T. viride* inoculation on growth and biomass resource allocation under P deficiency

Phosphorus deficiency results in reduced plant biomass, as evidenced by declines in both shoot and root dry weights (Tantriani et al., 2023). Consistent with these findings, our results indicated that shoot and root dry weight decreased significantly under treatments with insoluble P but increased following inoculation with *T. viride*. A positive correlation was observed between plant biomass and P content. Phosphorus deficiency leads to a higher allocation of carbohydrates to roots, reflected in an elevated R/S ratio and a redistribution of resources throughout the plant (Yan et al., 2019). Our study confirmed that the R/S ratio increased significantly under insoluble P conditions but was reduced following *T. viride* inoculation. Phosphorus deficiency prompted lower investment in shoot resources and higher allocation to roots, whereas *T. viride* inoculation reduced root resource allocation, except in the LP + T treatment. This allocation strategy of increasing root biomass, aimed at maximizing P uptake in P-deficient soils, is consistent across studies (Qi et al., 2022). Previous studies have reported reductions in the total root length, root surface area, and root volume in response to P deficiency (Mollier and Pellerin, 1999; Lopez et al., 2023). Our findings showed similar decreases in these root parameters under P deficiency and an inverse trend with *T. viride* inoculation in insoluble P treatments, leading to a greater resource allocation to shoots. Thus, *T. viride* inoculation makes resources more inclined to be allocated to the aboveground parts, which is beneficial to the production of more photosynthates in the aboveground parts, thus forming a benign transport of carbohydrates from the shoots to the roots.

## Conclusion

Our research established that P deficiency hampered the ability of photosynthetic pigments to capture light, thereby suppressing leaf photosynthetic electron transfer. Subsequently, the NPQ mechanism was activated to mitigate photooxidation resulting from P deficiency, leading to reduced PSII activity and lower CO_2_ assimilation rate, which significantly impair the photosynthetic capacity of *C. virgata*. In contrast, *T. viride* inoculation could improve the uptake of available P by *C. virgata* from insoluble forms, alleviate the photoinhibition caused by P deficiency, and promote biomass accumulation. Moreover, *T. viride* inoculation enhanced root development under insoluble P conditions. However, inoculation in low P conditions might provoke nutrient competition between *T. viride* and *C. virgata*, which could impede plant growth. In the presence of insoluble P, *T. viride* inoculation was associated with decreased PUE, PPUE, and leaf acid phosphatase activity in *C. virgata*. With the contribution of *T. viride*, *C. virgata* adjusted its resource allocation strategy, directing more resources toward shoot growth, resulting in a reduced R/S ratio, except for the LP + T treatment. Collectively, our findings suggest that *T. viride* can assist *C. virgata* in accessing available P from insoluble organic and inorganic sources, thereby enhancing growth. This insight is beneficial for researchers seeking to comprehend the mechanisms of forage grass in utilizing insoluble P in P-deficient soil and can offer strategies to reduce P fertilizer inputs.

## Data availability statement

The original contributions presented in the study are included in the article/Supplementary material, further inquiries can be directed to the corresponding authors.

## Author contributions

MS: Data curation, Formal analysis, Investigation, Methodology, Writing – original draft, Writing – review & editing. XL: Investigation, Writing – review & editing. XW: Funding acquisition, Methodology, Writing – review & editing. QZ: Investigation, Writing – review & editing. CM: Project administration, Writing – review & editing. XZ: Funding acquisition, Resources, Writing – review & editing.
