# Supplementary material for: Trichoderma viride improves phosphorus uptake and the growth of Chloris virgata under phosphorus-deficient conditions
Source: Front Microbiol. 2024 Jul 4;15:1425034. doi: 10.3389/fmicb.2024.1425034 (PMC11255847; doi:10.3389/fmicb.2024.1425034)
Supplement: Supplementary file 1 [file Data_Sheet_1.docx]

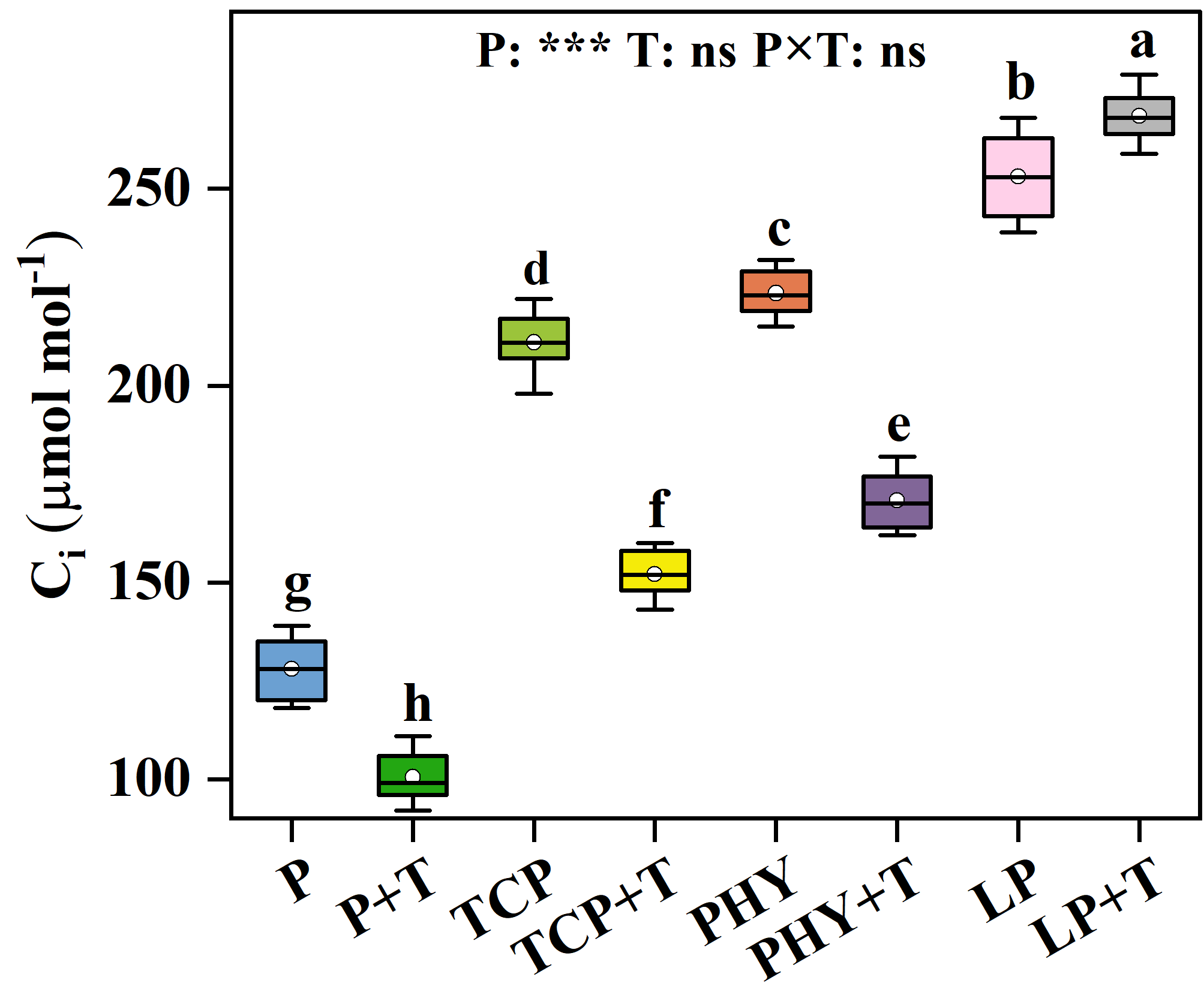


**Supplementary Figure 1** Effect of P deficient treatments on internal CO_2_ (C_i_). P, P was added as KH_2_PO_4_; TCP, P was added as tricalcium phosphate; PHY, P was added as calcium phytate; LP, P was added as 10 μM L^-1^ KH_2_PO_4_. ＋T, with *Trichoderma viride* (*T.viride*) inoculation. White dot is “Mean”; black diamond shape is “Outlier”; horizontal is “Median”; the top of vertical line is “Max” and the bottom of vertical line is “Min”. Different lowercase letters indicate significant differences in Duncan’s test (p< 0.05) (n=6). ***indicates significant difference extremely (p < 0.001). ns indicates no difference.

**Supplementary Table 1** Effect of P deficient treatments on LMA. Values are the means±S.E. (n=6) based on analyses by one-way ANOVAs followed by Duncan test. Different lowercase letters indicate significant differences between different treatments (p< 0.05).

| **Treatments** | **LMA** |
| --- | --- |
| P | 32.409±0.271a |
| P+T | 33.011±0.594a |
| TCP | 26.550±0.460c |
| TCP+T | 32.053±0.303a |
| PHY | 23.909±0.287d |
| PHY+T | 30.591±0.317b |
| LP | 21.420±0.286e |
| LP+T | 19.857±0.244f |
